# Involvement of interlukin-17A (IL-17A) gene polymorphism and interlukin-23 (IL-23) level in the development of peri-implantitis

**DOI:** 10.1038/s41405-024-00193-9

**Published:** 2024-02-28

**Authors:** Ehab Qasim Talib, Ghada Ibrahim Taha

**Affiliations:** Department of Basic Sciences, College of Dentistry, University of Baghdad, Baghdad, Iraq

**Keywords:** Peri-implantitis, Infection control in dentistry

## Abstract

**Background:**

Dental implantation has been practiced since ancient times and has gone through several stages. Dentists use dental implants to support dental prostheses such as crowns, bridges, dentures, face prostheses, or as an orthodontic anchor. Thus, the purpose of this study is to detect the role of the immune-genetic variation of IL-17A and related inflammatory cytokine (IL-23) in the initiation and progress of peri implantitis.

**Material and methods:**

This cross-sectional study included 80 subjects (15 peri-implantitis patients, 35 successful implants, and 30 healthy controls); their mean age was (43.91 ± 11.33) years. Blood samples and Peri-implant sulcus fluid (PISF) were collected from all subjects (patients with peri-implantitis, successful implants, and healthy controls) attending the Department of Oral and Maxillofacial Surgery in the Dental College Teaching Hospital, Baghdad University, Baghdad, Iraq. The blood sample detects gene polymorphisms in interleukin-17A by a polymerase chain reaction (PCR). An enzyme-linked immunosorbent assay (ELISA) was carried out to estimate the Peri-implant sulcus fluid (PISF) levels of interleukin-23.

**Result:**

The current study revealed an obvious significant elevation in the mean level of interleukin-23 in the peri-implantitis patient’s group more than its level in the successful implant and control groups (*P* < 0.05). In addition, the result showed that A/A genotype is associated significantly with peri-implantitis OR (95%confidence interval) =6.9 (1.7121 to 27.4638) folds increase risk of peri-implantitis) (*p* = 0.0065), while G/A genotype had OR 4.9 (0.9539–24.9394) folds increased risk of peri-implantitis, (*p* = 0.0572). But it was not statistically significant and G/G genotype had a one-fold increase risk of peri-implantitis.

**Conclusion:**

The increased level of inflammatory cytokine (interleukin-23) might add to the systemic inflammatory burden a predisposing factor, which may lead to impaired osseointegration and subsequent bone loss or implant failure. In addition, IL-17A gene polymorphism may play a role in peri-implant disease susceptibility, especially in persons carrying the rs2275913 A allele at a higher risk of developing peri-implantitits as compared with those carrying the G allele.

## Introduction

Dental implants are one of the most well-established and tried-and-true procedures in contemporary dentistry, and they are used to restore teeth that have been knocked out or lost. To support a dental prosthesis such as a crown, bridge, denture, or face prosthesis, or to operate as an orthodontic anchor, a dental implant is a surgical component that interacts with the bone of the jaw or skull [1]. It is also known as an endosseous implant or fixture. Titanium and its alloys are the most often used implant materials because they have a history of being effective in terms of their integration with the tissue around them. Titanium is also the most common implant material. Osseointegration is a biological process that must take place in order for materials such as titanium to successfully develop a close link with the bones [1, 2].

Osseointegration is “the direct structural and functional connection between ordered living bone and the surface of a load-carrying implant”. It allows load-bearing implants to merge with living bone. When host tissue fails to osseointegrate, an implant may fail [3]. Implant failures may be divided into early and late failures, or infected and noninfectious failures, based on their etiology. And may Early failures result from surgical trauma, premature loading, or bacterial infection, while late failures result from the implant’s inability to maintain osseointegration and may occur after prosthetics [3, 4]. However, tooth extraction and implantation cause an inflammatory response that usually leads to a smooth recovery and implant stability [5]. Localized inflammatory reactions usually contain and prevent harmful microorganisms, bring cells and their products to a lesion, and start the healing process. Inflammation involves several pathogenic pathways and cell types. Despite the discomfort, these reactions localize a disease and prevent it from spreading [6]. Pathogenic bacteria may inflame the supporting tissues of dental implants, like natural teeth [7]. The immune inflammatory response to bacterial stimuli, not bacterial biofilm, causes tissue damage in periimplantitis. Titanium implants cause tissue inflammation over time, according to multiple studies. This may produce mediators that cause local and systemic health concerns [8].

Peri-implant disease, mucositis, and titis are pathologies of dental implants. Immunological susceptibility to TH I and macrophage reactivity may cause titanium implant failure [5]. Peri-implantitis is known to cause bone loss and soft tissue irritation surrounding the implant [9].

This exceeds normal biological osseous remodeling. Implant survival and success have plummeted since this syndrome developed [1, 10, 11]. Interleukins and TNFs may affect osteonecrosis development, according to many studies [12].

Pro-inflammatory cytokines, including IL-1, IL-6, IL-8, TNF, and INF-, play pivotal roles in initiating acute phase reactions, modulating immune system activity, influencing central nervous system (CNS) responses, and regulating hormone release or repression. Their complex functions, though increasingly understood, highlight the dual nature of cytokines: while their induced responses are often beneficial, excessive production and activity can lead to detrimental effects such as hypotensive shock, organ failure, and death. To mitigate such risks, the body employs a sophisticated system of checks and balances, more intricate than endocrine mechanisms, to regulate individual cytokine production and activity [13]. A crucial aspect of this regulation involves CD4+ cells, which activate effector populations capable of causing inflammation [14]. Specifically, in periodontal gingival tissues, effector CD4 + T cell subpopulations, namely Th2 responses, are known to trigger the adaptive immune response. However, studies suggest that Th0 and Th1 responses are more prevalent in conditions such as periodontitis and periimplantitis, with the generation of IL-17 by Th17 cells, in conjunction with IL-23, shifting this paradigm [14, 15].

Interleukin-23 (IL-23), a cytokine crucial in the proliferation of the Th17 lineage, has been associated with immune-related tissue disorders and is particularly relevant to the Th17 pathways in periodontitis. This cytokine’s role in peri-implant lesions may indicate an ongoing inflammatory response, perpetuating the presence of IL-17-producing cells and thus maintaining a destructive inflammatory environment. Its significant role in peri-implantitis is attributed to its capacity to sustain chronic inflammation, potentially exacerbating the disease process [15, 16].

Interleukin-17 (IL-17), a unique pro-inflammatory cytokine belonging to a family with six isoforms and five receptors (IL-17RA, IL-17RB, IL-17RC, IL-17RD, and IL-17RSEF), is generated by CD4 + Th17 subsets and is known for promoting autoimmune and inflammatory disorders. The receptors of IL-17 may trigger immune-mediated inflammation, boosting pro-inflammatory cytokines, neutrophils, and monocytes. Located on 6p12.1, IL-17 is of particular interest due to its ability to increase the synthesis of RANKL, GMCSF, ICAM1, and prostaglandin E2, which are crucial in inflammatory processes [17].

IL-17’s significance stems from its role in inducing the production of other inflammatory molecules, contributing to the inflammation and bone destruction characteristic of the disease. Elevated levels of IL-17 have been associated with peri-implantitis, suggesting its involvement in the disease’s pathogenesis and leading to tissue destruction and bone resorption [15, 16].

Given the critical roles of IL-17 and IL-23 in sustaining and exacerbating inflammatory responses, understanding these cytokines is vital. Their potential as modifiable aspects of the immune response makes them attractive targets for therapeutic intervention, particularly in diseases like peri-implantitis where controlling the inflammatory milieu can have significant impacts on disease progression and patient outcomes.

IL-23 polymorphism or IL-17 expression might not be the most direct or established markers for bone resorption or destruction based on existing literature. Instead, the study has chosen IL-23 expression and IL-17 A gene polymorphism that have a well-documented association with the specific process under investigation.

The previous studies have consistently demonstrated the efficacy of the chosen markers in predicting bone resorption, it makes sense to align the current study with this body of evidence. Therefore, the aim of this study to detect the role of the immune-genetic variation of IL-17A and related inflammatory cytokine (IL-23) in the initiation and progress of peri implantitis.

## Materials

A total of 80 subjects were enrolled in this cross-sectional study and were divided into three groups:

**Peri-implantitis group:** consisted of 15 patients (10 male and 5 female), their mean age (39.93 ± 8.21) years.

**Successful implants group:** consisted of 35 subjects (20 males and 15 females), mean age was (47.33 ± 9.95) years with Successful dental implant.

**Control group:** consisted of 30 healthy subjects (19 males and 11 females), mean age was (41.67 ± 13.15) years). Were enrolled in this cross-sectional study.

This study is particularly significant as it represents a new method of investigation in dentistry to analyze the IL-17A SNP in relation to peri-implantitis.

Given this focus, the broad age distribution of study participants might be less relevant as a source of bias compared to studies where age-related factors significantly impact the outcomes. In genetic studies, the primary concern is often the genetic diversity rather than age.

The inclusion criteria of patients were required to have an unremarkable medical history, no known allergies, and no metabolic diseases. They also had no history of any antibiotic treatment for the prior 3 months, no use of anti-inflammatory drugs in the 6 months preceding the beginning of the study and no radiographic evidence of periodontal bone loss after a full-mouth radiographic periapical examination. The periodontal health of the patients was also evaluated using the plaque index (PI), gingival index (GI), pocket depth (PD) and bleeding on probing (BOP) by a dentist. The study protocol and informed consent forms were approved by the College of Dentistry, University of Baghdad.

### Sample size

It was calculated by Using G power 3.1.9.7 (Program written by Franz-Faul, Universitatit Kiel, Germany) With power of study = 92%, alpha error of probability = 0.05 with two-sided, and assuming the effect size as 0.40 (large) with three groups with all these conditions sample size is 73 subjects adding 10% as an error rate so the net sample size is 80 subjects. Effect size F are : Small = 0.1, medium = 0.25, large = 0.4.

### Insilico primer design

The source of all primers used in this study was Macrogen® (Korea). The name, sequence, and product size given is 541 bp with Sequence F- ATGACACCAGAAGACCTACAT, R- CCTGGATCTCCATAGTCAGAA.

### Sample collection

#### Blood collection

3 ml of venous blood were be drawn from patients under the aseptic technique and was added to the EDTA tube (1.5 mg/ml) then, kept at −70 °C were used the IL-17A gene detection by PCR.

#### Peri-implant sulcular fluid collection

90 min prior to the sample collection, patients were prepared for sample collection between 9:00 and 11:00 a.m. The patients were told to avoid eating and brushing their teeth. The areas that were being targeted were washed down with water, given some space using cotton rollers, and then given a little misting of air. For the purpose of collecting fluid samples from the test groups, “Perio Paper” was utilized. Following the removal of the supragingival plaque with the dry gauze, a normal paper strip was inserted into the sulcus and allowed to remain there for a period of thirty seconds. The part of the sample that was stained with blood was removed. The paper strips were immediately put in sterile Eppendorf tubes containing 0.5 ml of preservative (PBS), centrifuged at 3000 rpm for 10 min, and then kept at −80 °C until laboratory analysis [7]. This was done to maintain the integrity of the material and keep it in its original state. The evaluation and identification of IL-23 in the peri-implant sulcular fluid specimens were carried out with the use of enzyme-linked immunosorbent assay (ELISA)(BioTek/USA). (Catalog No: K0332151, BIOTECH, Korea).

#### Molecular detection of gene IL-17A polymorphism

DNA was extracted from patients with peri-implantitis, patients who had successful implants, and healthy control groups. Amplification of IL-17A was performed using a new set of primers in order to amplify 541 base pairs for usage in the sequencing section. Target SNPs will be involved in this area. In a PCR amplification reaction that was fifty microliters in volume, there was 25 µl of OneTaq (Catalog No: K0171, NEB®, England) master mix, 8 µl of DNA sample, 4 µl of each primer at a concentration of 10 pmol/l, and 9 µl of free-nuclease water. The reaction was carried out using the PCR conditions that are most suitable for this gene.

#### Gel electrophoresis

After PCR amplification, agarose gel electrophoresis was adopted to confirm the presence of amplification.

#### Agarose gel electrophoresis components

##### Tris-acetate EDTA buffer (10X solution)

Tris-Acetate EDTA solution was a “ready-to-use premixed solution that was used to dissolve agarose for gel preparation and as a buffer for electrophoresis after being diluted with DW to 1X strength (Carl ROTH® (Germany)”.

##### Red Safe™ nucleic acid staining solution

When attached to DNA or RNA, it exhibits green fluorescence, which is a stain for identifying nucleic acid in agarose gels (20,000×) Intron® (South Korea).

##### Preparation of agarose gel 2%

1. Using a volumetric cylinder, a volume of 1X TAE buffer containing 60 µl was measured.
2. A 1.2 mg was transferred of agarose powder, which corresponds to 2%, to a flask.
3. In order to dissolve the agarose, a 1X concentration of TAE buffer was poured over it.
4. The solution was brought to a boil in a microwave oven, and remained at that temperature until all of the gel particles had melted.
5. After waiting for the solution to reach 70 °C in temperature, 4 µl of the DNA staining dye RedSafe was added to the mixture.
6. The temperature of the solution has been brought all the way down to fifty degrees Celsius.
7. The agarose solution was put into the gel tray that had been prepared. At room temperature (between 20 and 25 °C), the solution was allowed to set for a period of 30 min.
8. Four hundred and sixty milliliters of 1X TAE were poured into the tank.

##### Molecular sequencing of IL-17A (rs2275913)

In order to determine the single nucleotide polymorphism, more than 40 µ of PCR product from each sample was sent to macrogen-Korea for sequencing using the Sanger technique. The Geneious Prime program was used to do an analysis of the sequence FASTA files, and the results were aligned to the RefSeq entry for the IL-17A gene, which has the accession number NG_033021.1.

### Statistical analysis

The Statistical Package for Social Science (SPSS) version 26(Cary, North Carolina, USA), and Microsoft Excel 2013 which was used to analyze the data. the present study’s data was carefully examined to determine if it was parametric or non-parametric using normality tests, as a result, appropriate statistical tests were employed Chi-Square, ANOVA and one-way ANOVA Post Hoc LSD test in addition to odd ratio test to Risk estimate statistics.

## Results

The age of all patients with peri-implantitis, successful implants and healthy control groups ranged from (21-68) years with a mean ± SD age was 43.9 ± 11.33 years.

Thirty-nine patients and control 31(38.8%) were female and 49 (61.3%) of males. As illustrated in Table 1.

**Table 1:** Demographic data of peri-implantitis patients, successful implant and healthy control groups.

Sex		Study groups			Total	*p* value
		Healthy control No. (%)	Implant successful No. (%)	Peri-Implantitis No. (%)		
Female		11 (36.7)%	15 (41.7)%	5(35.7)%	31 (38.8)%	0.888^**NS**^
Male		19 (63.3)%	20 (58.3)%	10(64.3)%	49 (61.3)%	
Total		30 (100.0)%	35 (100.0)%	15 (100.0)%	80 (100.0)%	
Age	Mean ± SD	41.67 ± 13.15	47.33 ± 9.95	39.93 ± 8.21	43.91 ± 11.33	0.052^**NS**^

The level of IL-23 in the peri-implant sulcular fluid of 15 patients diagnosed with peri-implantitis was measured and compared to the level of IL-23 in the peri-implant sulcular fluid of 35 successful implant group and 30 healthy individuals who served as the control group. The PISF level of IL-23 revealed a significant rise (*P* = 0.0001) in the group of patients who had peri-implantitis (609.1 ± 17.1). This was in contrast to the healthy control group and the successful implant group, which had levels of (120.0 ± 13.03) and (143.4 ± 12.3) respectively. In accordance with the findings shown in Table 2 and Table 3, respectively.

**Table 2:** PISF IL-23 levels in study groups and healthy.

ANOVA test	Peri-implantitis groups	Successful implant groups	Control groups	*p* value
Markers				
Peri-implant sulcular fluid level of IL 23 (pg/ml)	Mean ± SE	Mean ± SE	Mean ± SE	0.0001
	609.1 ± 17.1	143.4 ± 12.3	120.0 ± 13.03

**Table 3:** Comparison of IL-23 levels (pg/ml) in PISF between patients with peri-implantitis, successful implant group, and Healthy Control group.

Markers	Post-Hoc LSD one way ANOVA	Mean different	*p* value
IL-23 levels (pg/ml)	Peri-implantitis patients vs. Successful implant	465.1	0.0001
	Peri-implantitis patients vs. Healthy Control	489.1	0.0001
	Successful implant vs. Healthy Control	23.4	0.001

Table 4 shows the frequency of the GG, GA, and AA genotypes, of IL-17A gene in patients with peri-implantitis, successful implants, and Healthy control groups, which were (50.0%), (17.9%), and (5.6%) respectively in peri-implantitis patients compared to (31.3%), (53.6%), and (41.7%) successful implants, and compared to (18.8%), (28.6%), (52.8%) healthy controls. Significant differences in the frequency of the mutant homozygous genotype (AA) were observed between the two groups (*P* = 0.0001).

**Table 4:** Genotype frequency of IL-17A gene in peri-implantitis patient, successful implant, and healthy control groups.

Genotype	Peri-implantitis (*n* = 15)	successful implant (*n* = 35)	healthy control (*n* = 30)
AA	8 (50.0) %	5 (31.3)%	3 (18.8) %
GA	5 (17.9) %	15 (53.6)%	8 (28.6)%
GG	2 (5.6)%	15 (41.7)%	19 (52.8)%

Chi-square test = 172, *P* value = 0.0002.

**Fig. 1:**
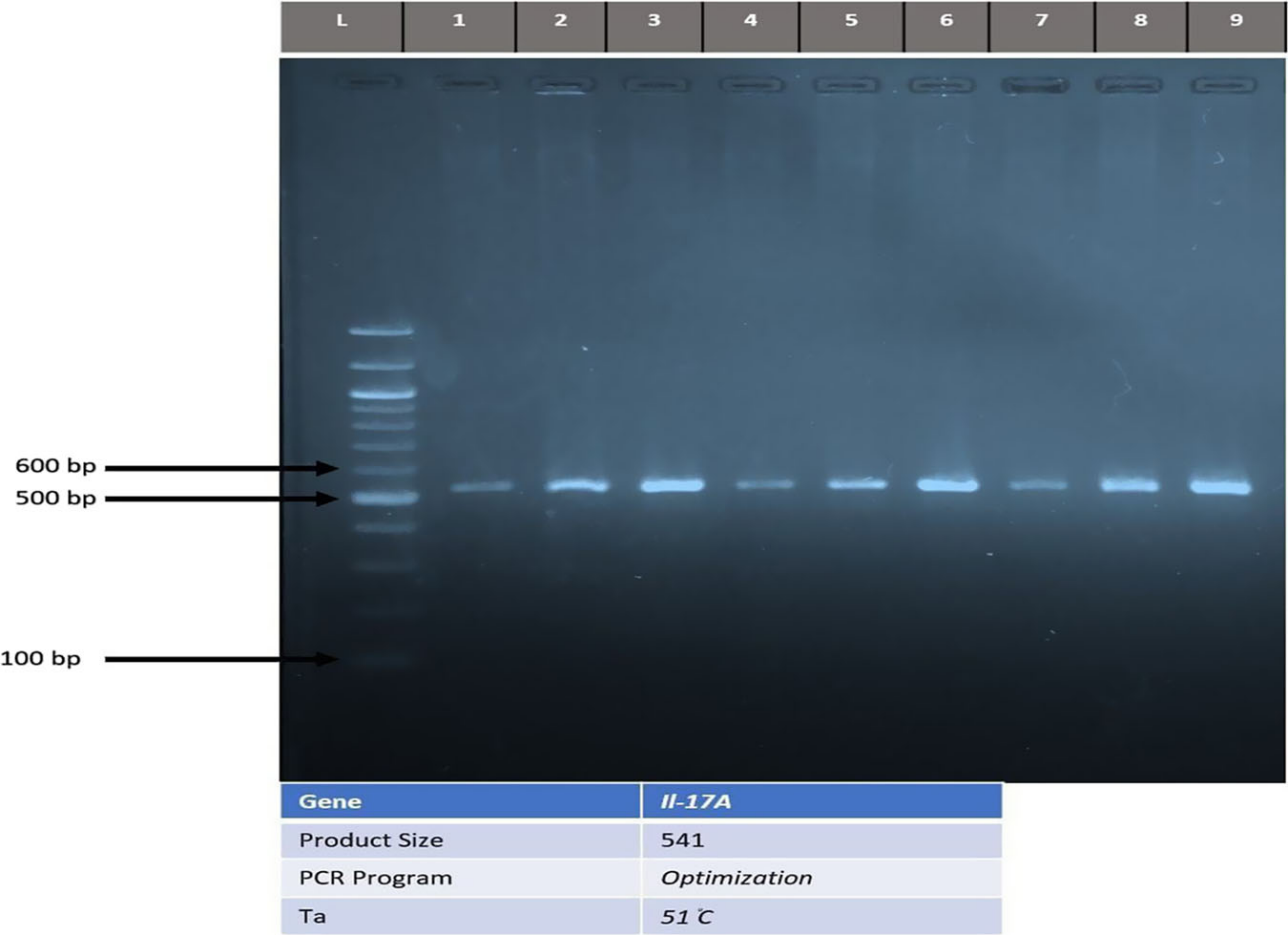
Agarose gel (2%) stained by RedSafe® and 75V electrophoresis for IL-17A gene. Lane 1 to 9 is giving product in an expected size 541 bp for patients group. L: DNA ladder (100 bp step), -C: Negative control.

**Fig. 2:**
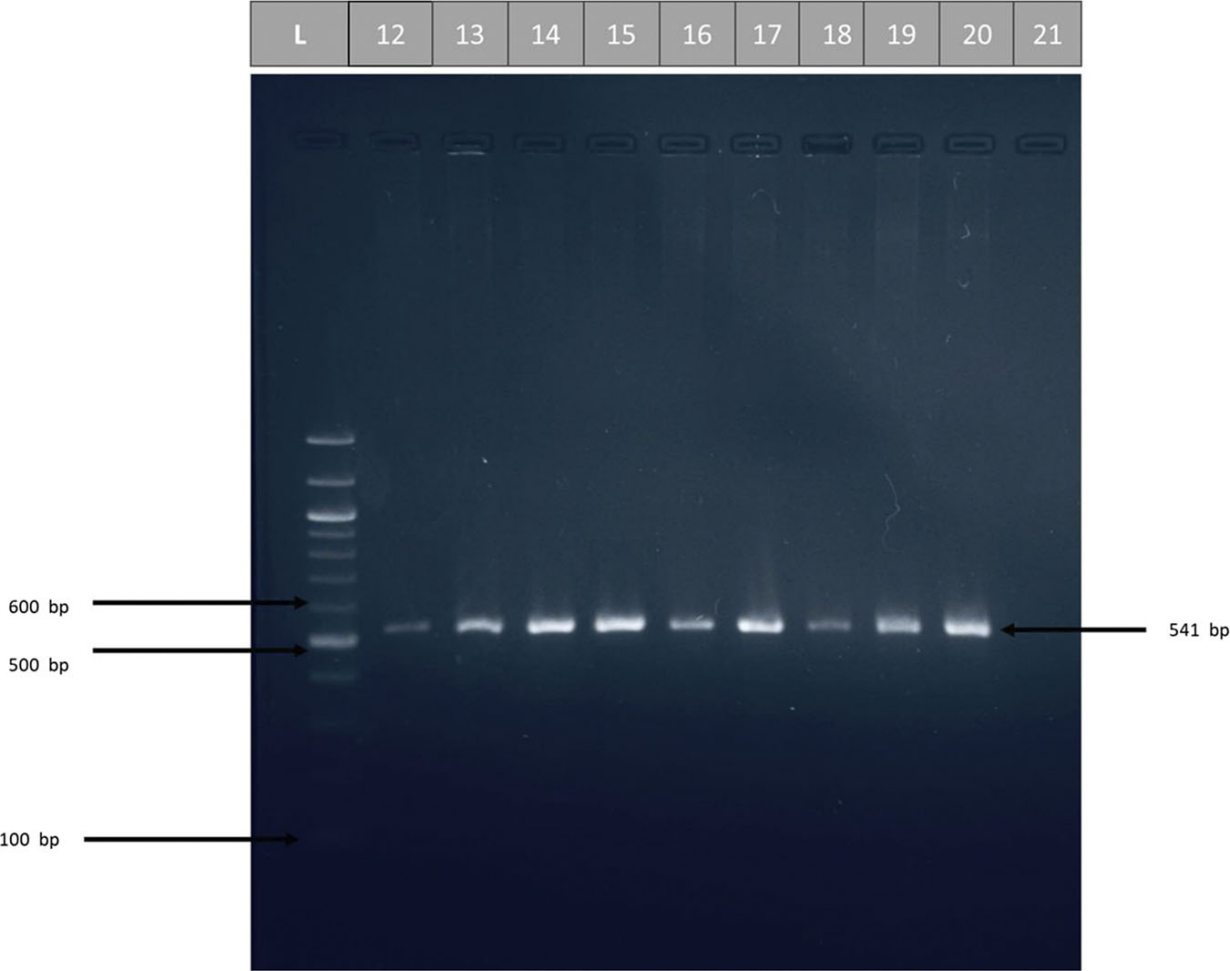
Detection of the PCR product DNA bands of IL-17A gene (541 bp) (Lane 12–20) for control group. The amplified fragments were separated by electrophoresis on a 2% agarose gel, stained with Red Safe dye at 75 volts/cm for 1 h. DNA ladder (100 bp step).

**Fig. 3:**
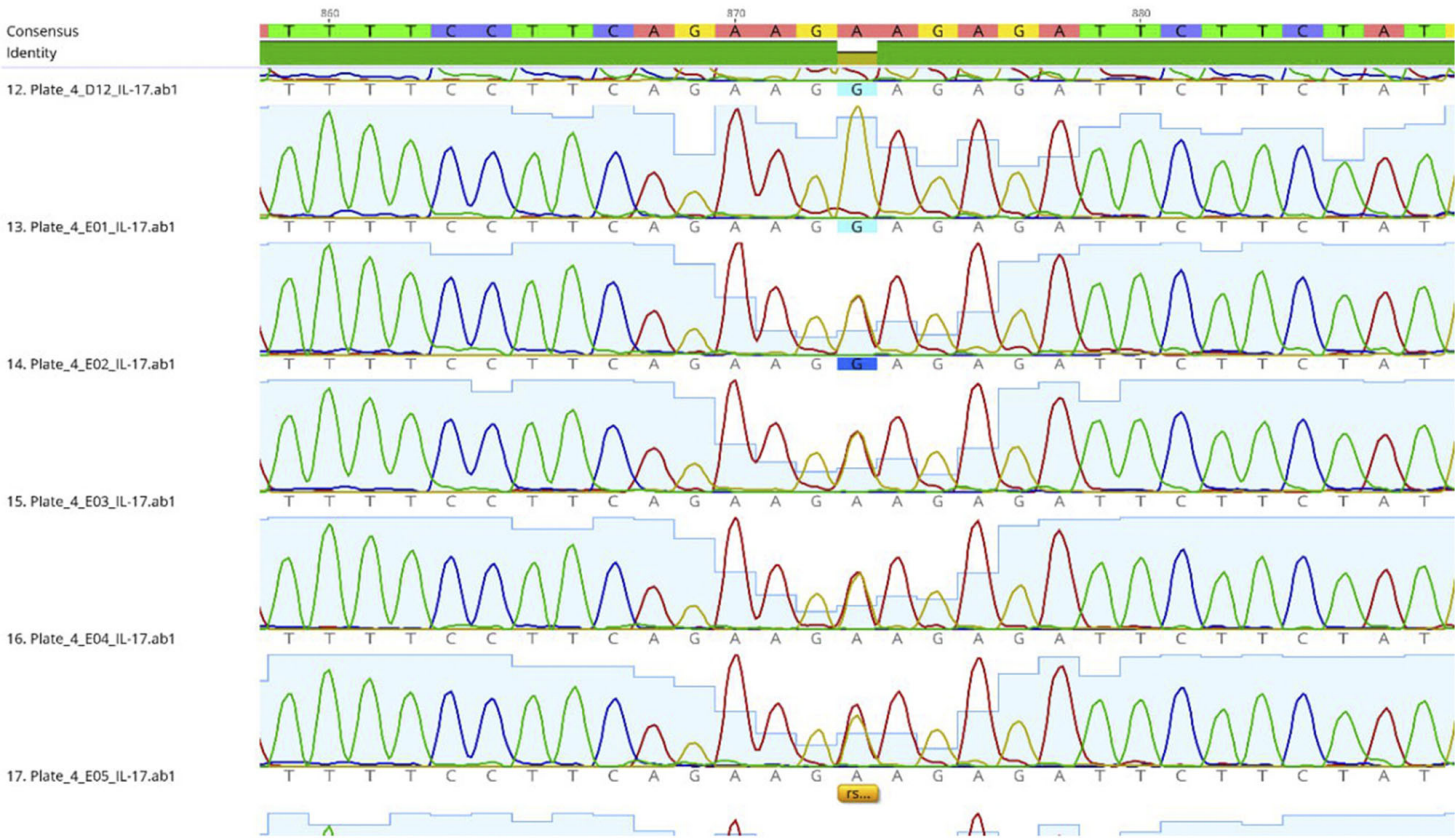
Analysis of sequence data from different samples related to IL-17A (G>A) polymorphism after pairwise alignment with IL-17 RefSeq (NG_033021.1), highlighting differences in the IL-17A gene sequence among individuals with different genotypes (GG, GA, AA). This visualization underscores the genetic diversity within the IL-17A gene and its potential implications for susceptibility and severity of peri-implant disease.

The difference was more prominent at the allele level, where the frequency of a mutant A allele in peri-implantitis group was more than in the successful implants and healthy control groups with a highly significant difference. Where the frequency of a mutant A allele in peri-implantitis group was more than in the successful implants and healthy control groups with a highly significant difference. As illustrated in the Table 4, Table 5, Figs. 1–3, respectively.

**Table 5:** The frequency of different alleles of IL-17A polymorphism in peri-implantitis patients and successful implants and healthy controls group.

Allele	Frequency			*p* value
	Healthy control	Implant successful	Peri-Implantitis	
G	46 (0.77)%	45 (0.64)%	9 (0.30)%	0.0001
A	14 (0.23)%	25 (0.36)%	21 (0.70)%

The comprehensive sequence analyses for the IL-17A (G>A) (Supplementary File 1) polymorphism across different genotypes (GG, GA, AA), are depicted in Figs. 3, S1, S2, and S3. These provide in-depth genetic insights crucial for understanding the variations observed in this study and underscore the genetic diversity pertinent to IL-17A’s role in disease pathology.

Table 6 shows that A/A genotype associated significantly with peri-implantitis (6.9 folds increase risk of peri-implantitis), while G/A genotype had 4.9 folds increased risk of peri-implantitis but it was not statistically significant and G/G genotype had onefold increase risk of peri-implantitis.

**Table 6:** IL-17A GENE polymorphism (197 G/A) (rs2275913) genotype in patients with peri-implantitis.

Risk estimate statistics (odd ratio test)		
Genotype	OR (95%CI)	*p* value
A/A	6.9 (1.7121 to 27.4638)	0.0065
G/A	4.9 (0.9539 to 24.9394)	0.0572
G/G	1.0	-

*OR* odd ratio, *CI* confidence interval.

Table 7 shows in the Healthy controls group, it was found that the mean ± SD value in these Subjects with the (GG), (GA) and (AA) genotype was (117.57 ± 9.32), (128.64 ± 13.83), (120.52 ± 11.57) respectively, there was significant differences between genotypes of IL-17A and PISF levels of IL-23. (*P* = 0.010). In the successful implant group, it was found that the mean ± SD value in these cases with the (GG), (GA) and (AA) genotypes was (135.05 ± 15.47), (148.56 ± 17.49) and (150.91 ± 20.36) respectively, there were no significant differences between genotypes of IL-17A and PISF levels of IL-23 (*p* = 0.0718). In the peri-implantitis group, it was found that the mean ± SD value in these cases with the (GG), (GA) and (AA) genotype was (609.02 ± 102.14), (609.09 ± 69.18) and (639.78 ± 176.51) respectively, there were no significant differences between genotypes of IL-17A and PISF levels of IL-23 (*p* = 0.921).

**Table 7:** Relationship of IL-17A gene polymorphism with PISF level of IL-23 in the three study groups.

Group	IL-17A gene polymorphism	Mean ± SD	ANOVA test *p* value
		IL-23	
Healthy control	GG	117.57 ± 9.32	0.010
	GA	128.64 ± 13.83	
	AA	120.52 ± 11.57	
Implant successful	GG	135.05 ± 15.47	0.0718
	GA	148.56 ± 17.49	
	AA	150.91 ± 20.36	
Peri-Implantitis	GG	609.02 ± 102.14	0.921
	GA	609.09 ± 69.18	
	AA	639.78 ± 176.51	

## Discussion

Dental implant failures are rare; however, they may occur during healing and after the first year of loading. Implant maintenance also causes issues [1]. Several longitudinal studies have shown 5–10-year survival rates of 85–95% [1, 3, 4]. Bacteria cause peri-implant tissue inflammation [13]. Local and systemic illnesses, drugs, systemic hormones, local cytokines, growth factors, and bone metabolism mediators might affect this response [18]. As in other infectious illnesses, bacteria can cause a peri-implant inflammatory disease, thereby the host responses towards these pathogens will determine the degree of disease development and severity [13]. Actually, the Peri-implantitis risk variables can be used to influence their therapy and prognosis. The clinical and radiological examinations are unfortunately insensitive to provide such information. Genetic biomarkers may be evaluated before the illness onset, and are recently preferable for many risks estimation. Nevertheless, the Biochemical indicators better track the illness onset, severity, and activity [7].

Regarding age and gender, this study found that there is no significant difference between peri-implantitis patients and successful implanted healthy controls. The mean age of the patients was 39.93 ± 8.21 years. This study agrees with Renvert et al. [19], Alsaadi et al. [20], and van Steenberghe et al. [21], who showed that age and gender have nothing to do with the peri-implantitis and suggest that the patient’s history, cardiovascular disease, and Smoking are the reasons for the peri-implantitis. In addition, Dreyer et al. [22] showed no association between age or gender in patients with maxillary implants and peri-implantitis. Moy et al. [23] reported that the patients’ gender, unlike age, did not have any impact on the success of the implant procedure. Age and gender are not factors that alone affect peri-implant disease and dental implant survival. Many Systemic disorders, such as osteoporosis, hypothyroidism, diabetes, hypertension, and heart ailments, A population or individual genetic polymorphism occurs when the DNA sequence differs.

Periimplantitis comes from the gingival plexus, a collection of blood vessels in the gingival corium. It is a physiological fluid and an inflammatory exudate that surrounds dental implants. The constituents of PISF have the potential to be used in the detection of subclinical changes in connective tissue remodeling, inflammatory cell recruitment, inflammatory mediators and biomarkers, and tissue metabolism [24]. Pro-inflammatory IL-23 is well recognized. Its ability to potently promote T-helper type 17 (Th17) proliferation is relevant to numerous inflammatory autoimmune responses and persistent infections. Antigens may also induce IL-23 and other cytokines [25, 26]. This study examined IL-23 levels in PISF from healthy individuals, peri-implantitis patients, and successful implant recipients using an ELISA kit. The patient group with peri-implantitis had a PISF IL-23 level that was statistically significantly greater (*P* = 0.0001) than the healthy control group and the successful implant group.

During peri-implant disease, the IL-23/IL-17 pathway may not work as it should, which could explain why IL-23 levels are high and gingival tissue isn’t protected. However, the cause of this dysregulation is unknown, requiring additional research. The present study was the same as those of Lester et al. [27], who found a significant increase in IL-23 in GCF and serum from patients with periodontitis and linked it to a faster rate of PPD progression. Unfortunately, no peri-implantitis research exists to compare.

Ohyama et al. [28], Rohaninasab et al. [29], and Althebeti et al. [30] reported elevated IL-17 and IL-23 in moderate to severe chronic periodontitis, especially around bone loss. According to their results, periodontal inflammatory disorders may activate and stimulate Th17 cells.

Schenkein et al. [31] and Borch et al. [32] studies on periodontal inflammation also confirm our study. Blood and GCF samples show elevated IL-17 and IL-23 levels. Local cytokine production increases systemic inflammation, which promotes periodontal tissue loss, they observed.

The current study agrees with Ju et al. [33], who found that IL-23 caused osteoclasts to form by directly increasing RANK in precursor cells and indirectly increasing RANKL on CD4 T cells, which led to bone resorption. Dutzan et al. [34] found that periodontal disease-affected tissues overexpressed IL-23 relative to healthy gingival tissues.

Jafarzadeh et al. [35] hypothesized that increasing IL-23 levels may exacerbate systemic inflammation. This may cause osseointegration failure, bone loss, and cardiovascular difficulties [36]. Cifcibasi et al. [37], found that periodontal therapy decreased IL-23. Santos et al. [38] showed variable results and little IL-23 change following treatment.

IL-23 in gingival crevicular fluid is related to relative attachment loss; hence, it may actively promote periodontal disease. Thus, it is a marker for periodontal inflammation during tissue degradation [39].

The amount of IL-23 in gingival crevicular fluid is linked to the loss of tissue around the implant, which shows that this cytokine causes inflammation.

DNA sequence differences cause genetic polymorphisms. SNPs, repeats, insertions, deletions, and recombination are examples. Viruses, radiation, and natural processes may produce genetic polymorphisms. Genetic mutations are DNA changes associated with sickness. Humans have the most SNPs. Understanding SNP functions may help us comprehend human phenotypic variability and the genetics of complex human illnesses [40]. It decided to analysis human blood samples to discover whether the mystery IL-17A cytokine’s genetic variation is linked to peri-implantitis. This is the first Iraqi dentistry study on the IL-17A SNP and peri-implantitis. The peri-implantitis group had a significantly higher mutant A allele frequency than the successful implants and healthy control groups.

Unfortunately, there were no studies concerning the IL-17A gene polymorphism in peri-implantitis to compare with the current study. Furthermore, a considerably higher rate of peri-implantitis was observed among people who had AA or GA genotypes, namely A allele-containing genotypes. So, the frequency of the homozygous (mutant) genotype AA in peri-implantitis was much higher than the frequency of the GG genotype and the G allele. These results agree with those of Saraiva et al. [41] and Chaudhari et al. [42], who reported the presence of allele A in the IL-17 gene polymorphism (-197A/G) for chronic periodontitis in the Indian and Brazilian populations. Patients who have this A allele could be more susceptible to the development of peri-implantitis or another peri-implant disease.

The current study disagrees with Kadkhodazadeh et al. [43]. In an Iranian community, patients with chronic periodontitis had a higher frequency of the IL-17A CC genotype than healthy controls. Corrêa et al. [44] indicated that periodontal disease was related to the IL17A G allele and GG genotype in the Brazilian population.

The findings of this study demonstrated that polymorphisms in IL-17 play a role in the occurrence of peri-implantitis. To date, many studies have investigated the controversial behavior of IL-17 subgroups (especially IL-17 A and F). However, an agreement has yet to be established concerning the function of this cytokine as a protective agent during local tissue inflammation or as a destructive factor.

Activated CD41 T cells are the main producers of IL-17. However, it has recently been discovered that neutrophils also release this cytokine [45]. It has been revealed that T lymphocytes are linked to bone deterioration via IL-17 production in rheumatoid arthritis [17]. IL-17 has been demonstrated to activate epithelial, endothelial, and fibroblastic cells to produce IL-6, IL-8, and PGE2 [46]. In addition, IL-17 stimulates RANKL production by osteoblasts [55].

Also, T-cell-derived cytokines, receptor activators of NF-kB ligand (RANKL), and receptor activators of NF-kB (RANK) have all been shown to be directly involved in bone metabolism. As IL-17 shares characteristics with IL-1 and TNF-a, it may regulate osteoclast-mediated bone resorption [47]. According to Espinoza et al. [48], who observed that the IL-17A 197A allele is associated with the production of an effective IL-17 and increased affinity for the nuclear factor of activated T cells (NFAT), which is a crucial regulator of the IL-17 promoter gene [48].

These findings of the current study suggest that the IL-17A (197A) allele was substantially linked to the peri-implantitis patient group. Subjects with the A allele are at 6.9 times greater risk of developing peri-implantitis than successful implant recipients and healthy control groups (odds ratio = 6.9, confidence interval = 95%). This result agrees with Chaudhari et al. [42], who reported that subjects with the A allele are at five times greater risk of developing localized aggressive periodontitis than healthy controls (odds ratio = 5.1, confidence interval = 95%).

Positive genotypes and peri-implant illness may have something to do with how IL-17 is released and controlled at the molecular level. Furthermore, Patients carrying the A (rs2275913 G/A) allele were associated with an increased risk for peri-implantitis or other peri-implant diseases as compared to people carrying heterogeneous type GA alleles or wild-type GG alleles. This may be in association rather than being a cause or having a direct effect. However, some investigations have revealed an elevated expression of IL-17 at the location of mild to severe periodontitis [25, 27, 34]. Schenkein et al. [31] observed a higher level of IL-17 in the systemic circulation of individuals with localized and widespread aggressive periodontitis.

## Conclusion

The current study supports the idea that the polymorphism of the IL-17A (-197 G/A) (rs2275913) genes may be a useful predictor for the pathogenesis of peri-implantitis. Furthermore, the IL-17A GENE polymorphism (197 G/A) (rs2275913) is associated with peri-implantitis susceptibility and may serve as a predictive and prognostic biomarker. The PISF level of IL-23 was significantly higher in the peri-implantitis patients than in successful implants and in the control group. This provides strong evidence that IL-23 plays a major role in the pathogenesis of peri-implantitis and could be causing devastating bone loss.

### Supplementary information

Supplementary Information

## Data Availability

The data that support the findings of this study are available from the corresponding author upon reasonable request.
