# Supplementary material for: Involvement of interlukin-17A (IL-17A) gene polymorphism and interlukin-23 (IL-23) level in the development of peri-implantitis
Source: BDJ Open. 2024 Feb 28;10:12. doi: 10.1038/s41405-024-00193-9 (PMC10899656; doi:10.1038/s41405-024-00193-9)
Supplement: Supplementary file 1 — Supplementary Information [file 41405_2024_193_MOESM1_ESM.pdf]

Supplementary File 1

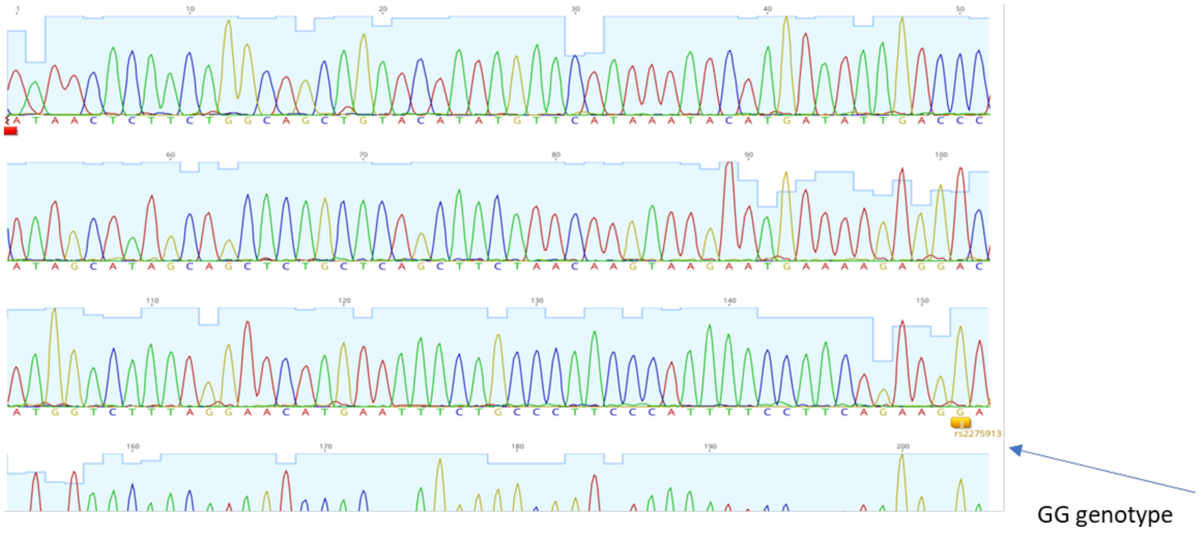

Figure S1: Showing sequence of analysis data of GG genotype of IL-17A gene

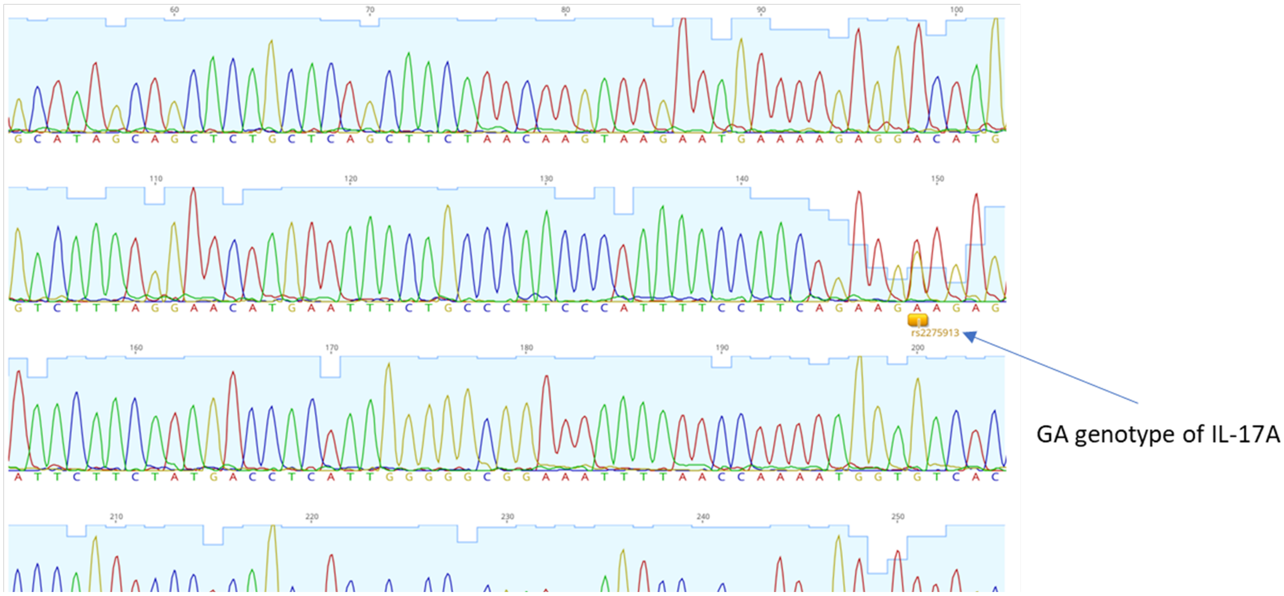

Figure S2: Showing sequence of analysis data of GA genotype of IL-17A gene

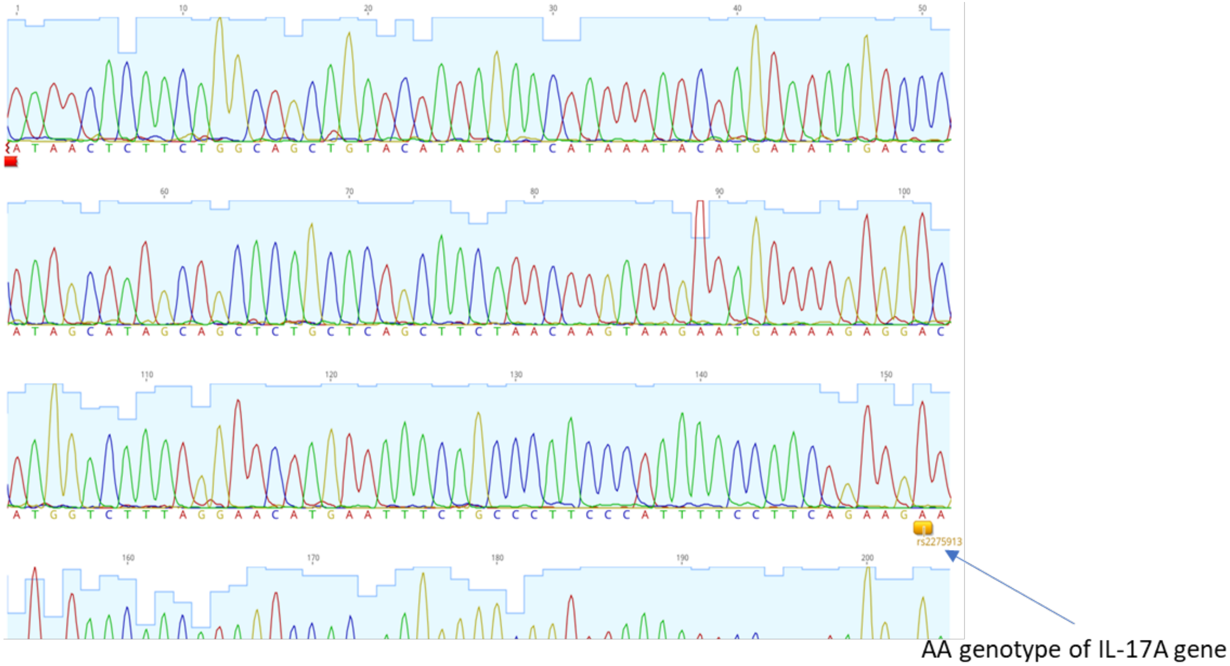

Figure S3: Showing sequence of analysis data of AA genotype of IL-17A gene
